# Altered infectivity, cell-cell fusion, and immune evasion of SARS-CoV-2 BA.3.2 and LP.8.1 variants

**DOI:** 10.1128/jvi.00016-26

**Published:** 2026-05-12

**Authors:** Pei Li, Yi-Min Zheng, Shan-Lu Liu

**Affiliations:** 1 Center for Retrovirus Research, The Ohio State University 2647, Columbus, Ohio, USA; 2 Department of Veterinary Biosciences, The Ohio State University 2647, Columbus, Ohio, USA; 3 Viruses and Emerging Pathogens Program, Infectious Diseases Institute,The Ohio State University 2647, Columbus, Ohio, USA; 4 Department of Microbial Infection and Immunity, The Ohio State University 2647, Columbus, Ohio, USA; University of Minnesota Twin Cities, Minneapolis, Minnesota, USA

**Keywords:** SARS-CoV-2, BA.3.2, LP.8.1, spike, cell-cell fusion, neutralization, glycosylation

## Abstract

The continued evolution of SARS-CoV-2 has yielded highly immune-evasive Omicron subvariants that challenge existing population immunity. In late 2024, a novel subvariant designated BA.3.2 emerged independently from the BA.3 lineage, carrying over 50 spike mutations, and representing a distinct evolutionary branch with unclear biological properties. Here, we characterize the BA.3.2 spike-mediated entry, fusogenicity, antigenicity, and neutralization in comparison to its parental BA.3- and JN.1-derived co-circulating LP.8.1 subvariants. Using lentiviral pseudotyping assays, we show that BA.3.2 exhibits significantly reduced infectivity in both 293T-ACE2 and CaLu-3 cells relative to BA.3, accompanied by attenuated spike-mediated cell–cell fusion. Despite its impaired entry, BA.3.2 evades neutralizing antibodies (nAbs) elicited by bivalent mRNA vaccination, Omicron BA.1-wave, and JN.1-wave infection, with a > 25-fold reduction in nAb titers compared to BA.3 in both bivalent mRNA vaccination and Omicron BA.1-wave cohorts. Antigenic mapping confirms remarkable divergences of BA.3.2 and LP.8.1 from the ancestral D614G, Omicron BA.3, and JN.1. Mechanistically, BA.3.2 acquires four new N-linked glycosylation sites in the N-terminal domain (NTD) and receptor-binding domain (RBD) of spike; disruption of which enhances viral infectivity and restores sensitivity to nAbs, suggesting a functional trade-off between immune evasion and spike functionality. Together, our findings define BA.3.2 as a highly immune-evasive variant with altered spike properties, shaped in part by glycan alteration. These results underscore the ongoing antigenic diversification of SARS-CoV-2 and highlight the importance of monitoring independent evolutionary trajectories outside the dominant JN.1 lineage.

**IMPORTANCE:**

The Omicron subvariant BA.3.2 has independently evolved from an early BA.3 lineage, carrying over 50 amino acid substitutions in its spike protein. Our study demonstrates that BA.3.2 exhibits markedly reduced infectivity and fusion activity but strong resistance to neutralizing antibodies elicited by vaccination or prior Omicron infection. We further show that newly acquired N-linked glycans in both the N-terminal and receptor-binding domains of BA.3.2 contribute to immune escape, while impairing spike-mediated entry. These findings reveal that glycan remodeling represents a key mechanism driving SARS-CoV-2 antigenic diversification and functional trade-offs between immune evasion and infectivity. Monitoring such independently evolving Omicron lineages is essential for understanding ongoing viral adaptation and for guiding future vaccine design.

## INTRODUCTION

The ongoing evolution of SARS-CoV-2 continues to generate variants with substantial antigenic and biological changes. In 2023, the emergence of the BA.2.86 lineage marked a significant evolutionary shift, characterized by over 30 spike protein mutations that distinguished it from the then-dominant XBB.1.5 variant (1). Subsequently, a BA.2.86-derived variant JN.1 defined by an additional L455S mutation in the spike rapidly rose to global dominance, with enhanced immune evasion capability (1–9). Since late 2023, JN.1 and its descendants, including KP.2, KP.3.1.1, XEC, and LP.8.1, have sequentially driven waves of infection worldwide, with the viral evolution largely continuing on the backbone of JN.1, resulting in further nAb escape (5, 7–15).

In November 2024 and January 2025, a highly divergent variant descended from the ancestral Omicron sublineage BA.3 was independently detected in South Africa (16). Subsequent genomic surveillance revealed continued diversification, with a derived variant, BA.3.2.2, detected 3 months later in South Africa (17, 18). By 9 November 2025, 86 BA.3.2 sequences from seven countries, including the Netherlands, Germany, Australia, Denmark, South Africa, the United Kingdom, and South Korea, had been reported to GISAID, representing 1.7% of globally available sequences in epidemiological week 45 of 2025, raising concerns about its potential for broader global dissemination (16, 17, 19). Although BA.3 itself originated in late 2021 and never achieved global dominance, the persistent detection of BA.3.2.2 in Western Australia (approximately 50% of clinical samples and 20% of wastewater sequences as of September 2025) indicates a slow but persistent wave, providing an evolutionary opportunity for additional adaptation (17, 20).

**Fig 1:**
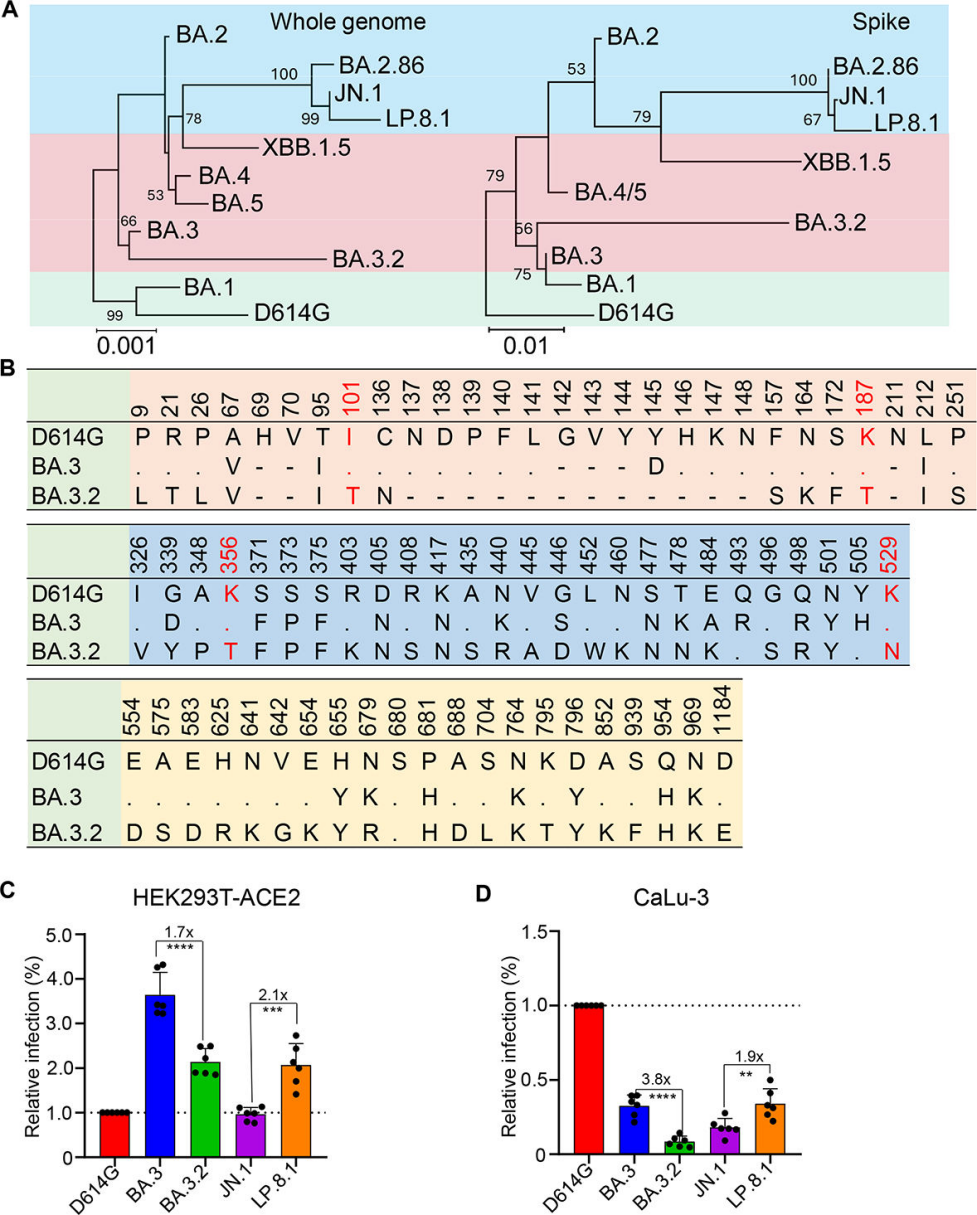
Relationship and infectivity of BA.3.2 and LP.8.1 in comparison to their ancestral variants, BA.3 and JN.1, in 293T-ACE2 and CaLu-3 cells. (**A**) Phylogenetic analyses of BA.3.2, LP.8.1, and key SARS-CoV-2 variant genomes and spike sequences. Maximum-likelihood phylogenetic trees were generated using MEGA 11 software, based on the alignment of either whole genome sequences (left) or spike amino acid sequences (right). Bootstrap values were calculated from 1,000 replicates, and the corresponding percentages are shown. Scale bars indicate the number of substitutions per site. (**B**) A schematic diagram illustrates the amino acid differences in the spike protein of BA.3.2 and BA.3 variants alongside D614G. Mutations are indicated by their specific amino acid changes, whereas identical residues across variants are represented by dots ("."). The orange box indicates the N-terminal domain (NTD) of the spike (S) protein, the blue box represents the receptor-binding domain (RBD), and the yellow box denotes the remaining regions of the spike protein. Red text indicates key mutations discussed in the manuscript. (**C and D**) Infectivity of lentiviral pseudotypes bearing BA.3.2, LP.8.1, or other spikes of interest. Infectivity was determined in 293T-ACE2 cells (**C**) and CaLu-3 cells (**D**). Bars in panels C and D represent means and standard deviation from six independent infections (*N* = 6). Significance was determined and displayed BA.3.2 relative to BA.3, JN.1 relative to LP.8.1: **P < 0.01, ***P < 0.001, ****P < 0.0001.

Phylogenetically, BA.3.2 is a divergent sublineage that branches from the early Omicron lineage BA.3 (18). It is distinct from BA.2-derived variants such as BA.2.86, JN.1, and LP.8.1, but clusters closely with BA.3 at both the whole-genome and spike protein levels (Fig. 1A and B). This suggests that BA.3.2 may retain its ancestral BA.3 features and exhibits different antigenic and functional properties compared to dominant recent variants, especially JN.1-derived subvariants. Remarkably, BA.3.2 carries over 50 spike protein mutations relative to BA.3 (Fig. 1B) and harbors a P681H mutation near the furin cleavage site, which is present in early alpha and Omicron BA.1/2 variants and has been shown to enhance S1/S2 processing and viral entry (21–23). In addition, BA.3.2 possesses four potential N-linked glycosylation sites in the N-terminal domain (NTD) and receptor-binding domain (RBD) (Fig. 1B), possibly contributing to immune evasion. Together, this magnitude of change could substantially alter its receptor engagement, cell entry pathways, and sensitivity to neutralizing antibodies (nAbs). These features also suggest that BA.3.2 may represent a novel and significant evolutionary trajectory for SARS-CoV-2, possibly foreshadowing another major antigenic shift.

In this study, we characterize the spike biology of BA.3.2. We examine its infectivity in HEK293T cells expressing human ACE2 and human low airway epithelial CaLu-3 cells, its fusogenicity, and its sensitivity to neutralization by sera from bivalent vaccine recipients and individuals infected during the Omicron BA.1 and JN.1 wave. In addition, we assess spike surface expression, furin-cleavage efficiency, and stability through S1 shedding assays. These parameters are compared to those of earlier as well as currently prevalent strains, including JN.1-descended LP.8.1. We also explore the role of newly acquired N-linked glycosylation sites in BA.3.2 by introducing T101I and T187K mutations to disrupt N-glycosylation motifs in NTD and T356K and N529K mutations in RBD. Together, our findings define the entry properties and immune escape potential of the BA.3.2 and LP.8.1 spike and have important implications for ongoing vaccine and therapeutic development as SARS-CoV-2 continues to diversify.

## RESULTS

### BA.3.2 spike mediates SARS-CoV-2 entry with reduced efficiency compared to parental BA.3

To assess the entry efficiency of BA.3.2, we generated lentiviral pseudotypes bearing the spike proteins of BA.3.2, its parental BA.3, and recent JN.1 and its descendant LP.8.1 variants. To control for possible differences in viral production, infectivity was evaluated using p24-normalized lentiviral particles, ensuring equivalent particle input across variants. Viral infectivity was evaluated in 293T cells stably overexpressing human ACE2 (293T-ACE2) and in the human lung epithelial cell line CaLu-3 (Fig. 1C and D). In 293T-ACE2 cells, both BA.3 and BA.3.2 exhibited increased infectivity than D614G, with 3.6-fold (*P* < 0.0001) and 2.1-fold (*P* < 0.001), respectively. Notably, BA.3.2 showed a 1.7-fold reduction in infectivity relative to BA.3 (*P* < 0.0001), placing it at a level comparable to the most recent LP.8.1 variants (Fig. 1C). LP.8.1 exhibited a modest 2.1-fold (*P* < 0.001) increase in infectivity compared to the ancestral JN.1 variant.

In CaLu-3 cells, we found that different from the ancestral BA.1 and BA.2 strain, which have been reported to exhibit a dramatically 5-fold to ~10-fold decreased infectivity (24, 25), BA.3 only showed a modestly reduced infectivity, that is, only a 3.1-fold decrease relative to D614G (*P* < 0.0001) (Fig. 1D). In contrast, BA.3.2 demonstrated a 12-fold reduction in infectivity (*P* < 0.0001) compared to D614G and a 3.8-fold (*P* < 0.0001) decrease relative to BA.3. JN.1 again showed ~5.5-fold (*P* < 0.0001) reduced infectivity relative to D614G, and LP.8.1 exhibited a ~ 1.9-fold (*P* < 0.01) increased infectivity relative to JN.1. Overall, BA.3.2 exhibited reduced infectivity in both CaLu-3 and 293T-ACE2 cells relative to BA.3, whereas LP.8.1 showed increased infectivity compared to JN.1 in both cell types, further highlighting the functional attenuation of BA.3.2 relative to BA.3.

### BA.3.2 and LP.8.1 exhibit decreased neutralization by COVID-19 vaccine, early Omicron sera, and JN.1 patient’s sera

**Fig 2:**
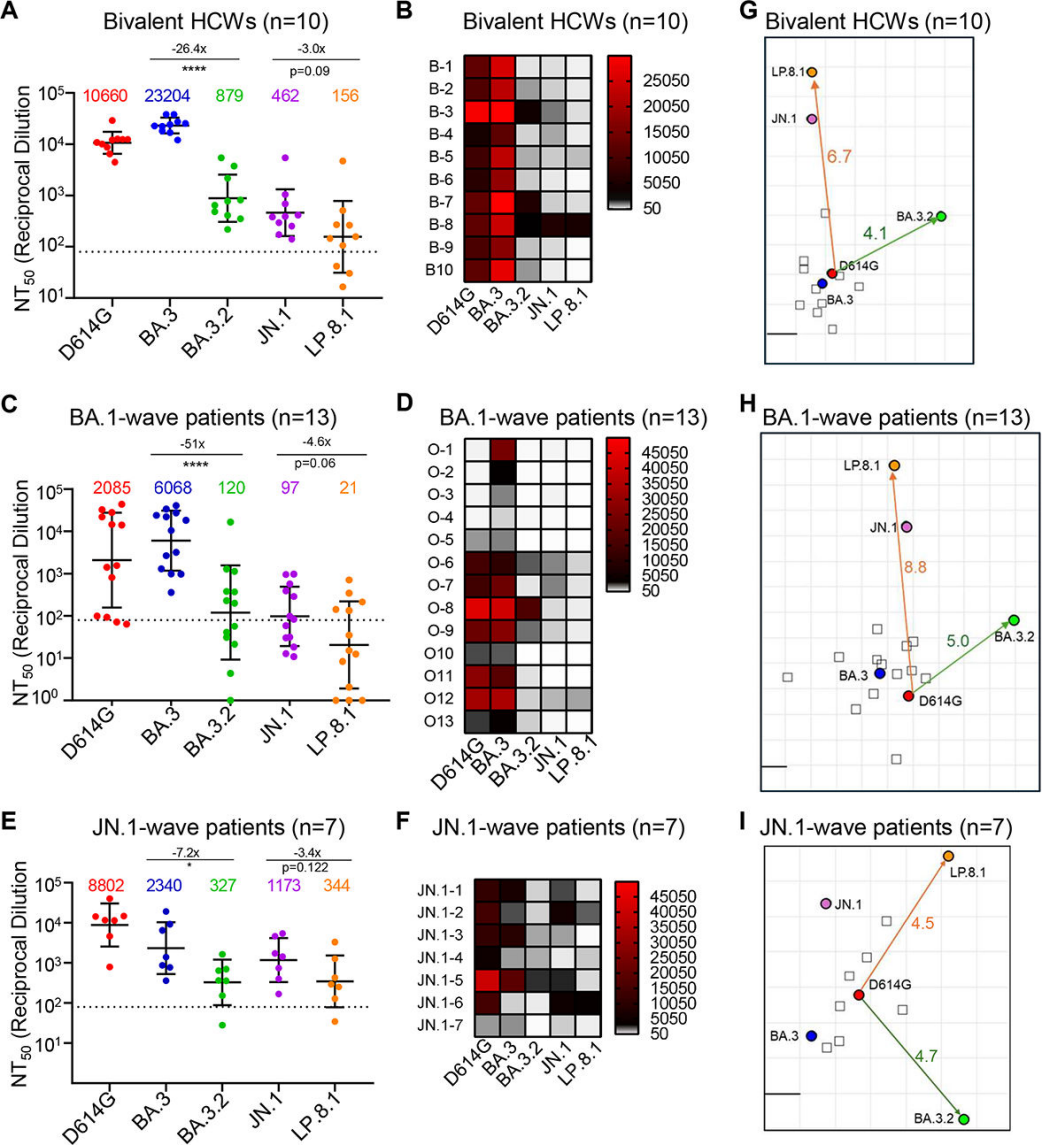
Neutralization and antigenic profiles of BA.3.2 and LP.8.1 against sera from bivalent-vaccinated healthcare workers, BA.1-infected individuals, and JN.1-infected individuals. NAb titers were determined against variants of interest in the sera of HCWs that received at least two doses of monovalent mRNA vaccine and a dose of bivalent (WT + BA.4/5) mRNA vaccine (*n* = 10) (**A and B**), individuals that were infected during the BA.1 wave of infection in Columbus, Ohio (*n* = 13) (**C and D**), and individuals that were infected during the JN.1 wave of infection in Columbus, Ohio (*n* = 7) (**E and F**). Bars represent means ± standard deviation. Plots in panels A, C, and E represent geometric mean nAb titers at 50% with standard errors. Geometric mean antibody titers are depicted at the top of the plots with fold changes relative to BA.3 or JN.1 above them. (**B, D, and F**) Heatmaps that depict the corresponding nAb values for each cohort, listed by individual samples. Significance was determined and displayed relative to BA.3 or JN.1 using log10 transformed values, unless otherwise indicated: ****P < 0.0001; *P < 0.05; ns, *P* > 0.05. (**G–I**) Antigenic analyses. The Racmacs program was used to plot relative antigenic distances between each spike antigen (circles) and serum sample (squares) for the bivalent-vaccinated HCWs (**G**), the BA.1-wave infected people (**H**), and JN.1-wave infected individuals (**I**). The scale bar represents one antigenic distance unit (AU), which is equivalent to about a 2-fold difference in nAb titer.

To evaluate the extent of immune evasion by BA.3.2, we examined neutralizing antibody (nAb) titers in sera from a cohort of healthcare workers (HCWs) at The Ohio State University Wexner Medical Center. All individuals had received at least two doses of a monovalent mRNA vaccine, followed by a bivalent booster encoding both wild-type and BA.4/5 spike proteins (*n* = 10) (Fig. 2A and B). We found that BA.3.2 exhibited a dramatic 26.4-fold reduction in nAb titers relative to its parental BA.3 (*P* < 0.0001), BA.3 showed an even 2.2-fold increased titer than D614G (*P* < 0.01). Notably, LP.8.1 demonstrated a 3-fold reduction in nAb titer compared to JN.1 (*P* = 0.09), in accordance with recent reports (26–28). Collectively, these data suggest that while both BA.3.2 and LP.8.1 are strongly immune evasive, although BA.3.2 is less than LP.8.1 in the context of bivalent mRNA vaccine-induced immunity.

We next assessed nAb titers in individuals infected during the BA.1 wave in Columbus, Ohio (*n* = 13) (Fig. 2C and D). Sera were collected between 1 and 7 days after hospital admission or clinical confirmation of BA.1 infection between 1 February and 3 March 2022, with the interval since last vaccination ranging from 64 to 367 days (29). In this group, we observed a 51-fold reduction in BA.3.2 neutralization relative to BA.3 (*P* < 0.0001), with the latter (BA.3) showing a 3-fold increased titer compared to D614G (*P* < 0.01). In contrast, both LP.8.1 and JN.1 were poorly neutralized, with titers near or below the detection threshold. Nonetheless, BA.3.2 appeared slightly less immune escape than LP.8.1 in this cohort. Overall, the findings from patients’ Omicron sera mirror the results from bivalent vaccine sera, collectively supporting the conclusion that BA.3.2 and LP.8.1 exhibit significant antibody evasion than their parental BA.3 and JN.1, respectively.

We also evaluated nAb titers in individuals infected during the more recent JN.1 wave in Columbus, Ohio (*n* = 7; sera collected between November 2023 and August 2024) (9) (Fig. 2E and F). As observed previously, all variants exhibited reduced neutralization titers relative to D614G, including JN.1. Notably, LP.8.1 and BA.3.2 showed the largest reduction, with 3.4-fold and 3.6-fold decreases in neutralization titer compared to JN.1, respectively; however, these differences did not reach statistical significance (*P* = 0.122 for LP.8.1; *P* = 0.09 for BA.3.2) due to the limited sample size (*n* = 7). These data show that both BA.3.2 and LP.8.1 possess significant immune-evasive properties in the context of JN.1-wave sera, with BA.3.2 exhibiting modestly greater immune evasion than LP.8.1.

### BA.3.2 and LP.8.1 are antigenically distinct from their parental BA.3 and JN.1 variants

To further evaluate the antigenic evolution of BA.3.2 and LP.8.1, we performed antigenic cartography using serum titers from the bivalent vaccine, BA.1-wave cohorts, and JN.1-wave cohorts described above (Fig. 2G, H and I). In bivalent vaccine and BA.1-wave cohorts, we found that LP.8.1 was most antigenically distant from D614G, exceeding its parental JN.1 (Fig. 2G and H). In contrast, in the JN.-1-wave cohorts, the antigenic distance of LP.8.1 from D614G was slightly shorter than that of BA.3.2 from D614G, measuring 4.5 versus 4.7 AUs, respectively (Fig. 2I). In all three cohorts, while BA.3 remained antigenically close to D614G, BA.3.2 was clearly distinct from D614G, BA.3, and LP.8.1 (Fig. 2G, H, and I). Altogether, these data highlight the profound impact of the more than 50 spike mutations in BA.3.2 relative to BA.3 on its antigenic profile and suggest that extensive spike diversification can substantially reshape antigenic relationships among contemporary variants, potentially influencing immune escape and vaccine effectiveness.

### BA.3.2 spike mediates reduced cell–cell fusion relative to BA.3

**Fig 3:**
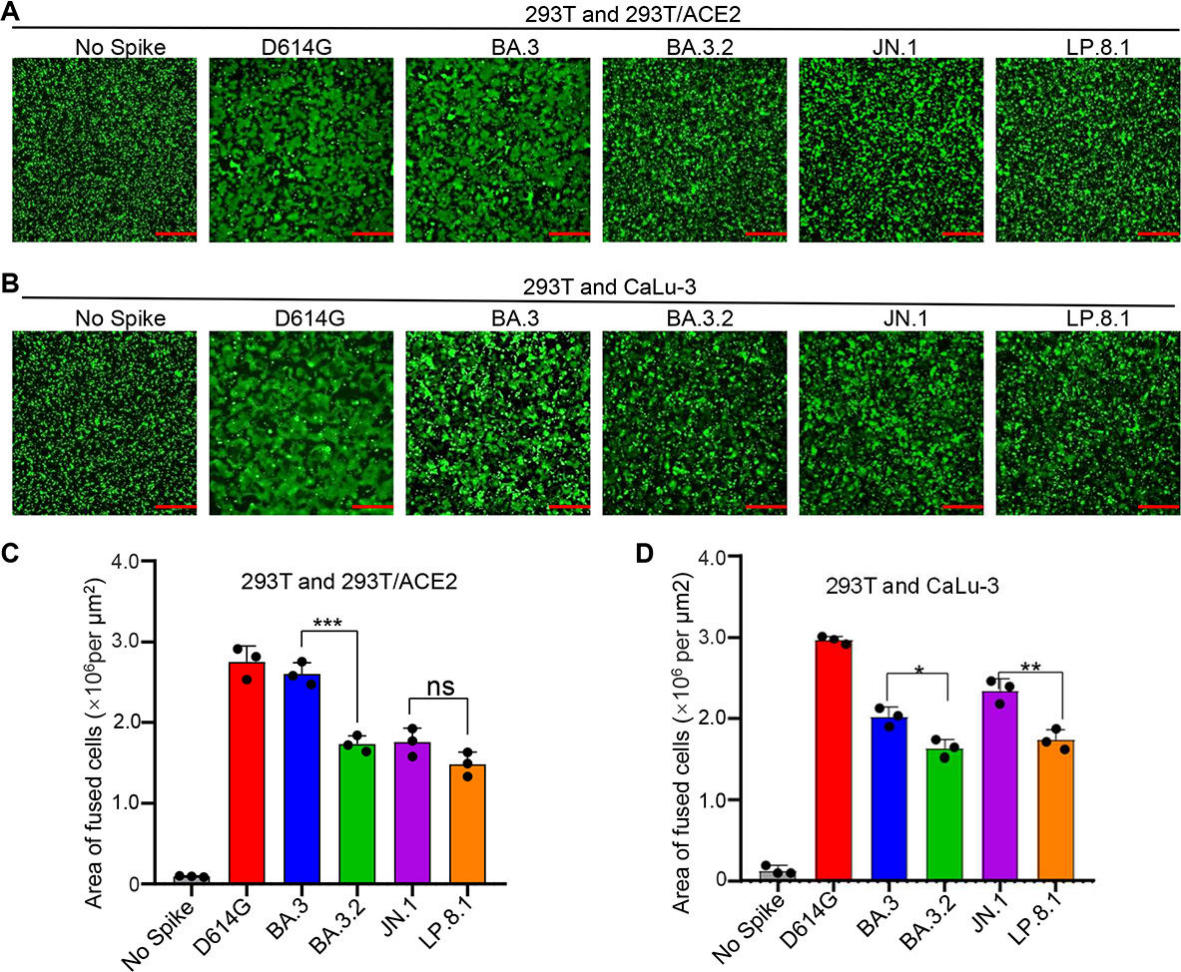
Cell-cell fusion activity of BA.3.2 and LP.8.1 compared with that of BA.3 and JN.1 variants. Fusion triggered between membranes by the spike proteins of interest was determined between 293T cells expressing the spike alongside GFP and 293T cells overexpressing ACE2, or CaLu-3 cells expressing an endogenous level of ACE2. Representative images of fusion are depicted for 293T-ACE2 (**A**) and CaLu-3 (**B**), and quantification of total areas of fusion across three images are represented for 293T-ACE2 (**C**) and CaLu-3 (**D**). The “No-Spike” condition was used to define fusion background, which displayed small, punctate GFP signals representing individual cells. Bars represent means with standard deviation, and significance was determined relative to ancestral variants as indicated: *P < 0.05; **P < 0.01; ***P < 0.001; ns, *P* > 0.05.

Given the extensive mutational divergence in BA.3.2, particularly near the furin cleavage site and within S2, we assessed spike-mediated membrane fusion using a cell-cell fusion assay. Spike-transfected 293T cells were co-cultured with either 293T-ACE2 or CaLu-3 cells, and fusion was imaged and quantified 4–6 h later. In 293T-ACE2 cells, BA.3 displayed cell-cell fusion capability comparable to D614G, whereas BA.3.2, JN.1, and LP.8.1 showed reduced fusion activity (Fig. 3A through D), consistent with previously reported Omicron BA.1/BA.2 lineages (24, 30, 31). In CaLu-3 cells, similar trends were observed, except that BA.3.2 exhibited a 1.2-fold reduced cell-cell fusion compared to BA.3 (*P* < 0.05), while LP.8.1 demonstrated a 1.4-fold reduction relative to JN.1 (*P* < 0.01). Overall, BA.3.2 and LP.8.1 displayed decreased fusogenicity compared to their parental variants, although the difference between JN.1 and LP.8.1 was less pronounced in 293T-ACE2 cells.

### BA.3.2 spike shows decreased processing and S1 shedding

**Fig 4:**
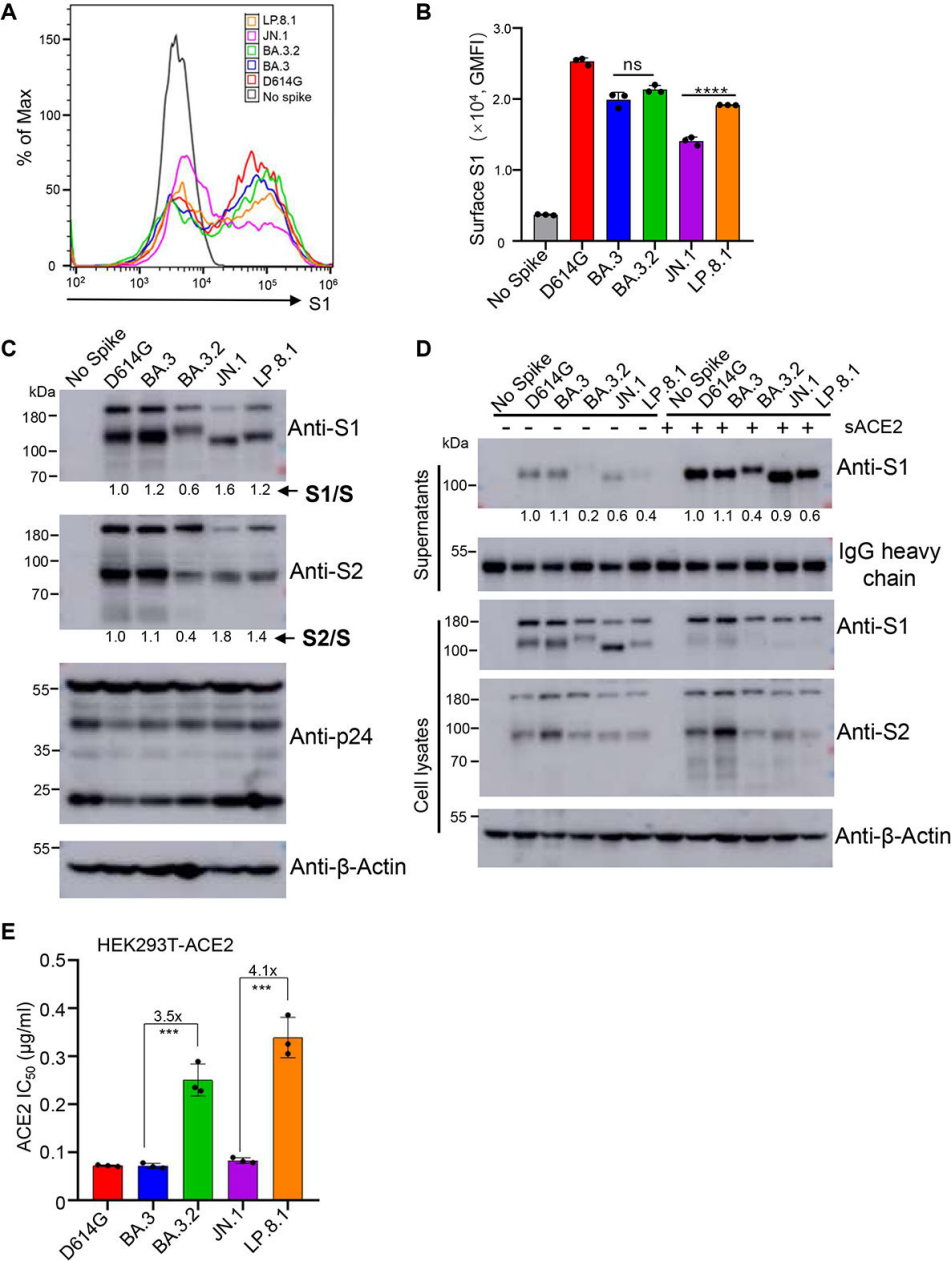
Surface expression, spike processing, and S1 shedding of BA.3.2 and LP.8.1 spikes. (**A and B**) The surface of 293T cells used to produce pseudotyped vectors was probed with anti-S1 antibody to compare surface expression between spikes of interest. Representative histograms depicting surface expression are shown (**A**), and geometric mean intensities (MFIs) of surface S1 are depicted (**B**) (*n* = 3). (**C**) Processing of spikes into S1/S2 subunits by furin was determined by lysing 293T cells used to produce pseudotyped viruses and probed by using anti-S1, anti-S2, anti-p24, and anti-β-actin antibodies. Relative ratios of S2/S or S1/S were quantified using NIH ImageJ, calculated by comparing to D614G. (**D**) HEK293T cells transfected with spike constructs of interest, without being treated with sACE2, were harvested; cell lysates were blotted with anti-S2, anti-S1, and anti-β-actin antibodies, and relative signals were quantified by NIH ImageJ by setting the value of D614G to 1.0. (**E**) Inhibition of spike-mediated entry by soluble ACE2 (sACE2). Lentiviral pseudoviruses bearing the indicated spike proteins were pre-incubated with serial dilutions of recombinant human sACE2 prior to infection of 293T-ACE2 cells. Infection was quantified by luciferase activity, and IC_50_ values were determined by nonlinear regression analysis. ****P < 0.0001; ***P < 0.001; ns, *P* > 0.05.

The cell-cell fusion activity of the spike protein is influenced by several factors, including proteolytic cleavage at the S1/S2 site, receptor engagement, and is directly associated with its surface expression levels (6, 25, 32). We therefore assessed their spike surface expression using flow cytometry of 293T cells used to produce lentiviral pseudotypes. All these post-Omicron variants exhibited decreased surface expression relative to D614G, with JN.1 showing the most pronounced decrease (Fig. 4A and B). While BA.3.2 displayed a comparable level of surface expression to BA.3 (*P* = 0.1), LP.8.1 showed a 1.3-fold increase relative to JN.1 (*P* < 0.0001) (Fig. 4A and B). Western blotting analysis of virus-producing cell lysates revealed that BA.3.2 spike had reduced furin cleavage efficiency compared to D614G and BA.3, as indicated by decreased ratios of both S1/S and S2/S (Fig. 4C, upper two panels). In contrast, JN.1 exhibited increased cleavage compared to D614G, consistent with our prior reports (6–9), while LP.8.1 showed decreased processing relative to JN.1. We also noted that the S1 band of BA.3.2 migrated more slowly than BA.3, possibly due to the acquisition of four new N-linked glycosylation sites (*see below*).

We next evaluated S1 shedding, which reflects RBD opening and subsequent destabilization of the S1–S2 interface, using spike-transfected 293T cells with or without soluble ACE2 (sACE2) treatment. As would be expected, sACE2 robustly stimulated S1 shedding from all spikes (Fig. 4D, upper panel), validating the effectiveness of the assay. Interestingly, BA.3.2 showed substantially reduced S1 shedding compared to BA.3, especially in the absence of sACE2 (Fig. 4D, upper panel), mirroring the decreased furin cleavage seen in producer cells (Fig. 4C). Of note, the S1 level in the cell lysate of sACE2-treated cells was lower than that of untreated, likely due to S1 shedding into the culture media (Fig. 4D, lower panel). In contrast, S2 levels in ACE2-treated cells were comparable to those of untreated cells. Surprisingly, LP.8.1 spike also showed reduced shedding compared to JN.1, especially in the absence of sACE2, consistent with reduced processing efficiency (Fig. 4D, upper panel), suggesting that LP.8.1 spike may be more conformationally stable compared to JN.1 (see Discussion). Consistent levels of anti-S1 antibody used to pulldown the shed S1, along with equivalent levels of spike and HIV-1 p24 expression, were observed, confirming the assay validity (Fig. 4C and D, middle and bottom panels).

Spike conformational dynamics are known to influence its engagement with the ACE2 receptor. We therefore assessed the sensitivity of these variants to inhibition by sACE2. Pseudoviruses were pre-incubated with increasing concentrations of recombinant human sACE2 prior to infection of 293T-ACE2 cells. We found that BA.3.2 exhibited a 3.5-fold higher sACE2 IC_50_ compared with BA.3 (*P* < 0.001), while LP.8.1 showed a 4.1-fold increase in sACE2 IC_50_ relative to JN.1 (*P* < 0.001) (Fig. 4E). These results suggest reduced ACE2 accessibility or engagement efficiency for BA.3.2 and LP.8.1 spikes relative to BA.3 and JN.1, respectively.

### Loss of N-linked glycosylation in spike recovers virus infectivity and neutralization

**Fig 5:**
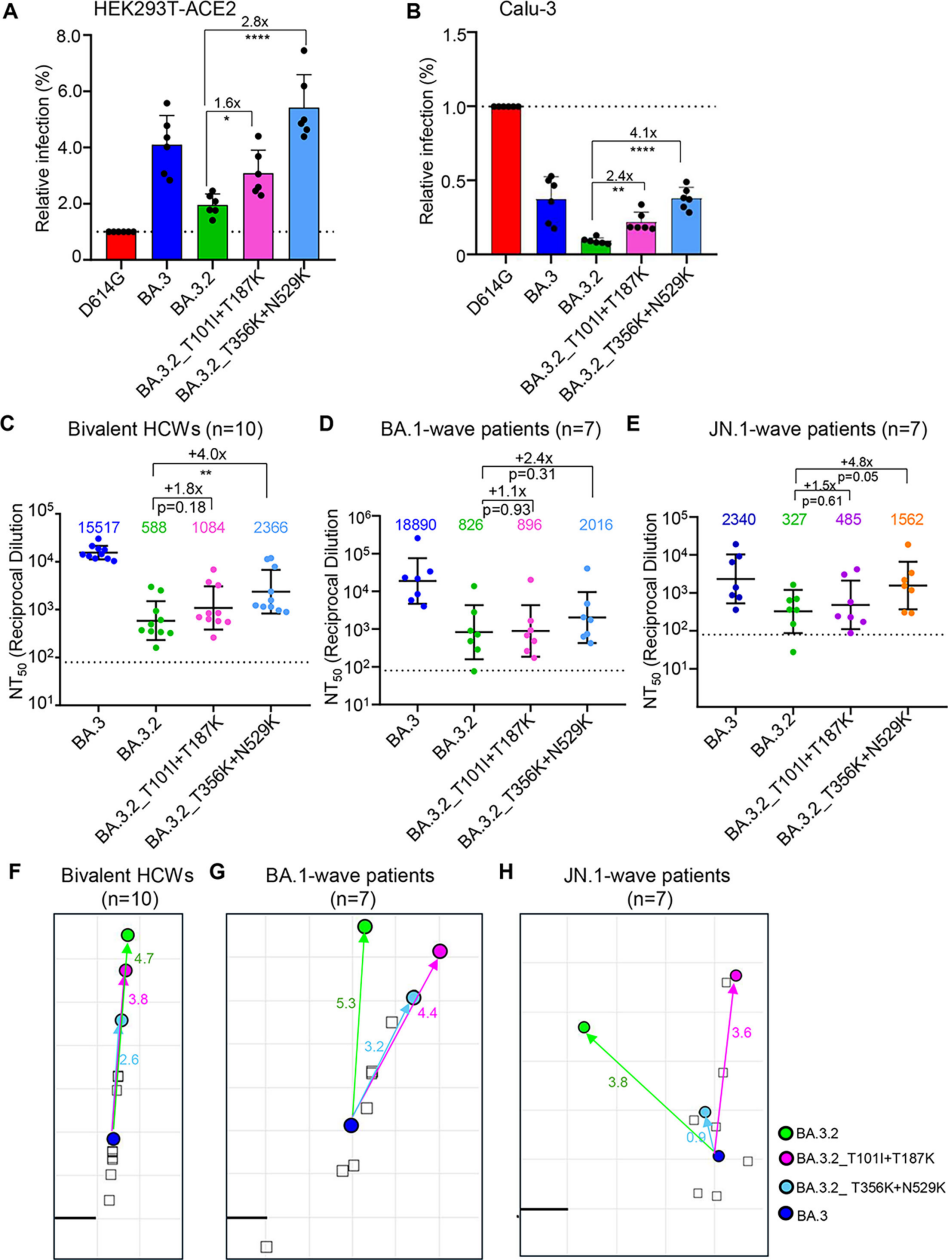
Effect of loss-of-glycosylation mutations in BA.3.2 on viral infectivity and neutralization by sera from bivalent-vaccinated healthcare workers and BA.1-infected individuals. (**A and B**) Infectivity of pseudotyped lentiviral vectors bearing variant spikes of interest was determined in 293T-ACE2 cells (**A**) and CaLu-3 cells (**B**). Bars in panels A and B represent means and standard deviation from six independent infections (*N* = 6). Significance was determined and displayed BA.3.2 relative to BA.3.2_T101I + T187K and BA.3.2_T356K + N529K: *P < 0.05; **P < 0.01; ****P < 0.0001. (**C–E**) Neutralization titers for HCWs that received at least two doses of monovalent mRNA vaccine and a dose of bivalent (WT + BA.4/5) mRNA vaccine (*n* = 10) (**C**), individuals that were infected during the BA.1 wave of infection in Columbus, Ohio (*n* = 7) (**D**), and individuals who were infected during the BA.1 wave of infection in Columbus, Ohio (*n* = 7) (**E**). Plots in panels C, D, and E represent geometric mean nAb titers at 50% with standard errors. Geometric mean antibody titers are depicted at the top of the plots with fold changes relative to BA.3.2. Significance was determined and displayed mutants relative to BA.3.2 using log10-transformed values, unless otherwise indicated: **P < 0.01; ns, *P* > 0.05. (**F–H**) Antigenic analyses. The Racmacs program was used to plot relative antigenic distances between each spike antigen (circles) and serum sample (squares) for the bivalent-vaccinated HCWs (**F**), the BA.1-wave infected people (**G**), and the JN.1-wave infected individuals (**H**). The scale bar represents one antigenic distance unit (AU), which is equivalent to about a 2-fold difference in nAb titer.

To elucidate the functional impact of the newly acquired N-linked glycosylation sites in BA.3.2, we disrupted these potential motifs (N-X-S/T, where X represents any amino acid except proline) by reverting the serine (S) or threonine (T) residues to their corresponding BA.3 residues, targeting sites in the NTD (T101I and T187K) and RBD (T356K and N529K), respectively. In 293T-ACE2 cells, these mutations markedly increased infectivity by 1.6-fold (*P* < 0.05) for the NTD mutant and 2.8-fold (*P* < 0.0001) for the RBD mutant (Fig. 5A). Similar increases were observed in CaLu-3 cells, with 2.4-fold for NTD and 4.1-fold for RBD mutations, respectively (Fig. 5B). Neutralization assays revealed a 1.8-fold (*P* = 0.18) and 4.0-fold (*P* < 0.01) increase in nAb titers from bivalent vaccinee sera against the NTD and RBD mutants, respectively, relative to BA.3.2 (Fig. 5C). Similar trends of increase were observed for both glycan-deficient mutants in a subset of seven BA.1-wave and seven JN.1-wave patient sera, although the differences were not statistically significant due to the limited sample size (*n* = 7) (Fig. 5D and E).

To assess how these glycan-deficient mutations affect spike antigenicity, we performed antigenic cartography using sera from the same bivalent vaccine and BA.1-wave cohorts described above. In the bivalent vaccine cohort, removal of NTD glycans (T101I + T187K) reduced the antigenic distance of BA.3.2 relative to BA.3 from 4.7 to 3.8 antigenic units (AUs), whereas removal of RBD glycans (T356K + N529K) resulted in a distance of 2.6 AUs (Fig. 5F). Comparable patterns were observed in the BA.1-wave and JN.1 cohort, where the BA.3.2 RBD glycan-deficient mutant exhibited a shorter antigenic distance to BA.3 than the BA.3.2 NTD mutant, that is, 3.2 AUs versus 4.4 AUs in BA.1-wave patients, and 0.9 AUs versus 3.6 AUs in JN.1-wave patients, respectively (Fig. 5G and H). Collectively, these findings indicate that loss of RBD glycans has a stronger effect on restoring antigenic similarity to BA.3 than loss of NTD glycans, underscoring the dominant role of RBD-associated glycans in shaping immune evasion.

**Fig 6:**
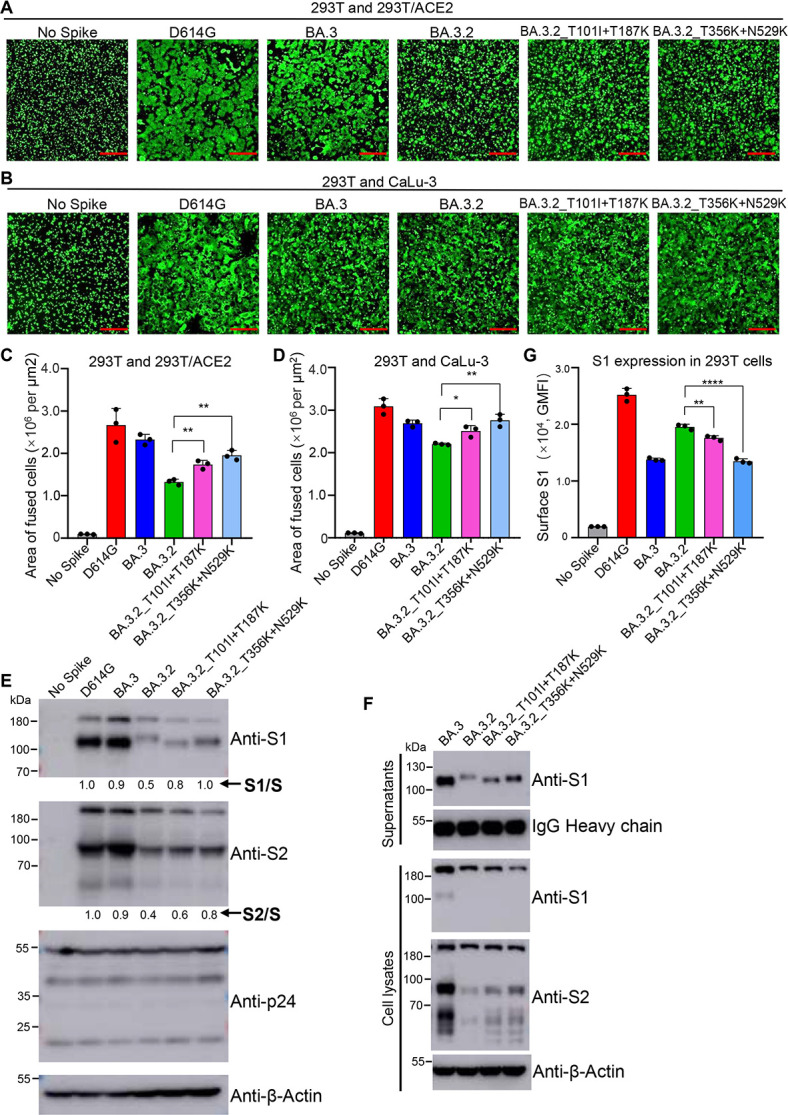
Cell-cell fusion and S1 shedding of BA.3.2 spike glycosylation mutations. Fusion was determined between 293T cells expressing the spike plus GFP and 293T cells overexpressing ACE2, or CaLu-3 cells expressing an endogenous level of ACE2. Representative images of fusion are depicted for 293T-ACE2 cells (**A**), and CaLu-3 cells (**B**), and quantification of total areas of fusion across three images are represented for 293T-ACE2 cells (**C**) and CaLu-3 cells (**D**). Bars represent means with standard deviation; significance was determined relative to ancestral variants as indicated : *P < 0.05; **P < 0.01; ****P < 0.0001. The surface expression of Spike of interest in 293T cells used to produce pseudotyped vectors was determined by probing with an anti-S1 antibody. (**E**) Processing of spikes into S1/S2 subunits by furin was determined by lysing 293T cells used to produce pseudotyped viruses and probed by using anti-S1, anti-S2, anti-p24, and anti-β-actin antibodies. Relative ratios of S2/S or S1/S were quantified using NIH ImageJ, calculated by comparing to D614G, and are displayed under corresponding blots. (**F**) HEK293T cells transfected with spike constructs of interest, treated or untreated with sACE2, were harvested and stained with an anti-S1 antibody. Cell lysates were blotted with anti-S2, anti-S1, and anti-β-actin antibodies. (**G**) Geometric mean intensities (MFIs) of surface S1 are depicted (*n* = 3). *P < 0.05; **P < 0.01; ****P < 0.0001.

The above results suggest that mutations at the glycosylation site in BA.3.2, particularly those in the RBD, can critically influence the biology of spike, including fusogenicity and possibly the conformational stability. We thus performed cell-cell fusion and spontaneous S1 shedding assays using transient transfection assays of 293T cells. In cell-cell fusion assays, we observed that the NTD glycan-deficient mutant (T101I + T187K) exhibited a modest (1.3-fold) but consistent increase in fusogenicity when cocultured with 293T-ACE2 cells (*P* < 0.01), while the RBD glycan-deficient mutant (T356K + N529K) showed a greater 1.5-fold increase (*P* < 0.01) (Fig. 6A and C). Similar results were observed in CaLu-3 cells, with the RBD mutant showing almost a comparable level of fusion to that of BA.3 (Fig. 6B and D). Western blotting analysis of the viral producer cells revealed increased spike processing for both mutants, again with a more pronounced effect observed for the RBD glycan-deficient spike (Fig. 6E), consistent with the marked restored fusion activity. S1 shedding assays demonstrated increased levels of S1 release for both mutants, especially the RBD glycan-deficient mutation (Fig. 6F). Notably, across all immunoblots, the loss-of-function glycosylation mutants showed faster electrophoretic mobility, consistent with the expected decrease in molecular weight (Fig. 6E and F). Flow cytometric analyses of the viral producer cells showed reduced surface S1 levels for these mutants, inversely correlating with their enhanced S1 shedding (Fig. 6G). Altogether, these data support the notion that the newly acquired N-linked glycosylation sites in BA.3.2, especially those in the RBD, facilitate immune evasion but compromise infectivity, reflecting a trade-off between antibody escape and receptor engagement.

## DISCUSSION

In this study, we characterized the recently emerged SARS-CoV-2 subvariant BA.3.2, which harbors over 50 amino acid substitutions in its spike protein compared to its parental BA.3 lineage. Our analysis demonstrated that BA.3.2 has undergone substantial antigenic and functional divergence from its ancestral BA.3, resulting in decreased efficiency in entry and cell-cell fusion (Fig. 1 and 3) but enhanced immune evasion (Fig. 2). Biochemical data show that the spike protein of BA.3.2 is less efficiently processed into S1 and S2 subunits compared to BA.3 (Fig. 4), which explains in part the impaired cell-cell fusion and infectivity. The increased sACE2 IC_50_ observed for BA.3.2 and LP.8.1 further suggests reduced receptor accessibility during viral entry. While sACE2 inhibition does not directly measure binding affinity or spike conformational state, these findings are consistent with a model in which these variants may spend more time in a closed spike conformation, which could limit ACE2 engagement and thereby reduce entry efficiency, contributing to immune evasion (Fig. 1 and 4). However, direct structural or biophysical analyses will be required to definitively establish possible conformational differences among these spikes.

We observed that LP.8.1 also exhibited reduced S1 shedding despite showing enhanced S1/S2 cleavage compared with its ancestral JN.1 (Fig. 4). This finding suggests that the LP.8.1 spike may be structurally more stable than that of JN.1. Noticeably, LP.8.1 contains several key amino acid substitutions relative to JN.1, including the N-terminal domain deletion delS31, which our previous studies have previously shown to stabilize the spike and reduce S1 shedding (7, 8). The increased structural stability of the LP.8.1 spike likely contributes to the observed reduction in S1 shedding, which may underline its decreased fusogenicity and enhanced immune evasion, as seen in BA.3.2.

Our infectivity analyses in cultured CaLu-3 cells, which model the human lower airway epithelium, have revealed a dynamic pattern along the evolutionary trajectory from the ancestral D614G to Omicron BA.1, BA.2.86, and the more recent JN.1 sublineages (4, 6–9, 24, 25). BA.1 displayed markedly reduced infectivity in CaLu-3 cells compared with pre-Omicron variants, especially Delta, consistent with its diminished fusogenicity and shift toward endosomal entry (25, 30, 31, 33). This reduction in TMPRSS2-dependent entry has been associated with the attenuated lower respiratory tract infection and decreased pathogenicity characteristic of early Omicron lineages (31, 34–36). While BA.2.86 partially restored infectivity in CaLu-3 cells, it gained a modest recovery of spike–host membrane fusion efficiency and partial adaptation to TMPRSS2 usage (25, 37). Subsequent JN.1 sublineages exhibited reduced infectivity in CaLu-3 cells, similar to BA.1 and BA.2 (6–9, 24, 31, 34). In line with this trend, BA.3.2 again showed decreased infectivity compared with BA.3 (Fig. 1), correlating with its reduced spike processing, cell–cell fusion, and S1 shedding (Fig. 3 and 4). Overall, this pattern of changes, from an initial decrease, partial rebound, to subsequent decline in CaLu-3 infectivity, suggests that Omicron evolution continues to favor the upper airway tropism over the lower respiratory tract, although detailed *in vivo* studies using authentic SARS-CoV-2 variants are needed to confirm these findings.

Our neutralization and antigenic cartography data from bivalent vaccine recipients, Omicron BA.1-wave, as well as JN.1-wave convalescent sera, reveal a pronounced escape phenotype for BA.3.2 and LP.8.1 (Fig. 2). These three serum panels were selected to represent distinct immune histories, including vaccine-induced immunity and immunity shaped by early Omicron BA.1 waves and more recent JN.1 waves. Together, they allow us to assess antigenic drift across different stages of population-level immunity and determine whether the escape phenotype is broadly maintained across diverse immunological backgrounds. BA.3.2 in particular showed more than a 25-fold reduction in nAb titers relative to its ancestral BA.3 in bivalent sera, with an even larger reduction in convalescent sera from BA.1-infected individuals. Our neutralization results somewhat differed from those reported by Guo et al. and Zhang et al. (26, 38). In sera from bivalent-vaccinated healthcare workers and BA.1-wave patients, our studies found that LP.8.1 exhibited greater immune escape than BA.3.2. However, in JN.1-wave sera, BA.3.2 showed comparable or modestly greater immune evasion relative to LP.8.1, in agreement with other reports. Some of these differences could reflect variations in immune background among the serum panels analyzed, particularly the use of early BA.1 and more recent JN.1 lineage exposed sera, highlighting the potential impact of cohort-dependent immunity on antigenic ranking, consistent with its modest antigenic shift (26, 38). Of note, BA.3.2 displays reduced infectivity in our *in vitro* assays. Thus, to achieve efficient transmission comparable to JN.1, BA.3.2 would likely need additional adaptive changes that enhance receptor engagement and entry efficiency, thereby balancing immune escape with optimal infectivity. These patterns mirror observations for highly evasive JN.1-descendant subvariants (6–9, 27, 28), including LP.8.1, although direct comparisons using authentic viruses would provide definitive evidence.

Consistent with other Omicron subvariants (5, 30, 33, 35, 36, 39), we found that BA.3.2 exhibits reduced spike-mediated cell–cell fusion relative to D614G. We also showed that BA.3.2 displays even lower fusion than its ancestral BA.3 in both 293T-ACE2 and CaLu-3 cells, with levels comparable to LP.8.1 (Fig. 3). This reduction in fusion capacity is closely associated with impaired entry, spike processing, and reduced S1 shedding (Fig. 1 and 4), supporting a model in which the pronounced immune evasion of BA.3.2 comes at the cost of reduced entry efficiency and spike activation potential. These observations, once again, underscore a recurring evolutionary trade-off in SARS-CoV-2, where adaptations that enhance antibody escape can compromise viral entry and cell–cell spread, shaping the virus’s fitness landscape in the context of host immunity (6–9, 28, 40–47). We noted that Zhang et al. reported no significant difference in fusion between BA.3.2, LP.8.1, and BA.3, and this discrepancy could reflect differences in assay design, sensitivity, and timing of detection. In our system, fusion was evaluated at an early time point (4–6 h) following co-culture of spike-expressing and receptor-expressing cells, whereas Zhang et al. assessed syncytium formation after 18 h. Given that membrane fusion is a time-dependent and cumulative process, differences in incubation duration may contribute to the variation in observed outcomes.

Glycosylation plays a critical role in coronavirus spike biology by influencing viral entry, infectivity, and immune evasion (7, 8, 48, 49). In this study, we investigated the impact of newly acquired N-linked glycans in BA.3.2 by selectively disrupting motifs in the NTD and RBD. Loss of these glycans significantly enhanced infectivity and cell–cell fusion in both 293T-ACE2 and CaLu-3 cells (Fig. 5), indicating that glycan acquisition can impede spike–receptor interactions. Removal of RBD glycans produced a more pronounced increase in infectivity and restored neutralization sensitivity, highlighting their dual role in sterically restricting ACE2 binding and shielding key antibody epitopes (7, 8). While our data are consistent with altered glycan positioning or shielding, we were unable to directly measure the glycan occupancy at individual sites and therefore cannot completely rule out alternative structural or conformational effects. Future studies using site-specific glycoproteomic approaches will be required to definitively confirm glycan occupancy at these positions. Interestingly, despite reduced entry efficiency, BA.3.2 maintains substantial antibody escape (Fig. 2 and 5), reflecting an evolutionary trajectory that prioritizes immune evasion over transmissibility, a strategy that may shift under future population-level immunity. From a vaccine design perspective, these observations highlight the importance of accounting for glycan-mediated shielding when developing next-generation immunogens aimed at eliciting broadly protective antibody responses. Given the global reliance on spike-based vaccines and antibody therapeutics, monitoring of glycan modifications, epitope shifts, and antigenic distances is essential for informing pandemic preparedness strategies.

## MATERIALS AND METHODS

### Cell lines

Human epithelial kidney cells (293T, ATCC, RRID: CVCL_1926) and 293T cells overexpressing human ACE2 (293T-ACE2) (BEI Resources, RRID: CVCL_A7UK) were maintained in DMEM (Sigma Aldrich, Cat #11965-092) supplemented with 10% fetal bovine serum (Thermo Fisher, Cat #F1051) and 0.5% penicillin/streptomycin (HyClone, Cat #SV30010). Human lung adenocarcinoma cell line CaLu-3 cells were maintained in EMEM (ATCC, Cat #30-2003) supplemented with the same components. To passage, cells were washed in phosphate-buffered saline then detached using 0.05% Trypsin +0.53 mM EDTA (Corning, Cat #27106). Cells were maintained at 37°C with 5.0% CO_2_.

### Plasmids

Spike was cloned into the pcDNA3.1 plasmid backbone with a FLAG tag at the C-terminal end of the coding sequence except D614G and BA.3.2, which have a FLAG tag at both N- and C-terminal ends. BA.3.2 was synthesized and cloned into pcDNA3.1 using KpnI/BamHI restriction enzymes by GenScript Biotech. JN.1 and LP.8.1 spike were generated through site-directed mutagenesis from BA.2.86 (synthesized by GenScript), and each of the other variants was generated by site-directed mutagenesis from JN.1. Pseudotyped HIV-1 vectors are based on the pNL4-3-inGluc originally received from David Derse (NIH) as previously described (50).

### Study cohorts

All data were collected from our cohorts under approved IRB protocols as follows: Bivalent mRNA vaccination: 2020H0228, 2020H0527, and 2017H0292; Omicron/BA.1-wave patients: 2020H0527; JN.1-wave patients: 2020H0175. The first human cohort was the Ohio State University Wexner Medical Center healthcare workers (HCWs) that received at least two doses of monovalent WT mRNA vaccine and a dose of the bivalent (WT + BA.4/5) mRNA booster vaccine (*n* = 10) (8, 9, 25). All individuals were administered two homologous doses of mRNA vaccine—five received Moderna and five received Pfizer. Nine individuals received a third dose of vaccine (4 Moderna, 5 Pfizer), while one individual did not receive a third dose. Five individuals were administered the Pfizer formulation of the bivalent vaccine, while five received the Moderna formulation. Blood was collected between 23 and 108 days post-bivalent dose administration. Individuals ranged from 27 to 46 years old, with a median of 37 years; five males and five females were recruited.

The second cohort of samples was obtained from 13 patients hospitalized during the Omicron wave (February 1 to March 3, 2022) (24). Positivity for SARS-CoV-2 infection was confirmed via RT-PCR, and the infecting variant was determined through sequencing of nasopharyngeal swabs and next-generation sequencing. This cohort included four unvaccinated and nine vaccinated patients. Among the vaccinated individuals, three had received two doses, and six had received three doses of the vaccine. Ages ranged from 32 to 78 years, with a median of 62; five females and eight males were recruited to this cohort.

The last cohort was patients at the OSU Wexner Medical Center who were either admitted to the ICU during the JN.1 wave of infection in Columbus, OH (11/23/2023–8/11/2024) (*n* = 4), or collected from first responders and household contacts in the STOP-COVID cohort who were symptomatic during that time period (*n* = 3) (9). RT-PCR was used to confirm COVID-19 positivity, and the infecting variant was determined using next-gen sequencing (Artic v5.3.2, IDT, Coralville, IA, and Artic v4.1 primers, Illumina, San Diego, CA). Ages ranged from 35 to 77, with a median of 51 years; four females and three males were recruited to this cohort (7).

### Phylogenetic analysis

Whole-genome and spike protein amino acid sequences of SARS-CoV-2 variants were aligned using the ClustalW algorithm implemented in MEGA 11 software (51–53). Phylogenetic trees were constructed using the maximum-likelihood method in MEGA 11. The amura-Nei model was used for nucleotide sequences and the Jones–Taylor–Thornton (JTT) model for amino acid sequences. Bootstrap analysis with 1,000 replicates was performed to evaluate the confidence of branching.

### Lentiviral pseudotype production and infectivity

Pseudotyped lentiviruses were produced via polyethyleneimine transfection (Transporter 5 Transfection Reagent, Polysciences, Cat #26008-5) of 293T cells with a 2:1 ratio of pNL43-inGluc vector and spike (54). Viruses were collected 48 and 72 h post-transfection and used to infect target cells 293T-ACE2 and CaLu-3 cells. To measure readouts, equal volumes of infected cell media and *Gaussia* luciferase substrate (0.1 M Tris pH 7.4, 0.3 M sodium ascorbate, 10 µM coelenterazine) were combined, and luminescence was determined by a Cytation 5 Imaging Reader (BioTek). These readings were taken 48 and 72 h post-infection.

### Virus neutralization assay

Viral infectivity was determined for each variant and normalized to ensure that comparable infectious viral particles were used for this assay (54). Sera from the various cohorts were serially diluted to final dilutions 1:80, 1:320, 1:1,280, 1:5,120, 1:20,480, and one no-serum well for each individual sample. sACE2 was diluted to 10, 2.5, 0.625, 0.156, 0.039, and 0 µg/mL. Equal volumes of normalized vector were added to the serially diluted sera and incubated for 1 h at 37°C. The mixtures were then used to infect 293T-ACE2 cells and relative infectivity determined at 48 and 72 h post-infection as described above. Anti-nucleocapsid (N) antibodies were not measured in this study, limiting our ability to distinguish recent infection-induced responses from pre-existing immunity. Neutralization titers at 50% were calculated via least squares fit nonlinear regression using GraphPad v10 (San Diego, CA) with values normalized to the no serum/antibody control.

### Antigenic cartography analysis

Racmacs v1.1.35 was used to generate the antigenic maps (55). Briefly, instructions detailed on the GitHub entry (https://github.com/acorg/Racmacs/tree/master) were used to run the program in R (Vienna, Austria). Raw neutralization titers are input into the program, where they are then log2-transformed and plotted in a distance table. This distance table is then used to perform multidimensional scaling and plot the individual serum samples (squares) and antigens (circles) in two-dimensional space. These plots are scaled by antigenic distance units (AU), where 1 AU = about a 2-fold difference in nAb titer. Program optimizations were kept on default, and maps were exported using the “viewmap” function and labeled using Microsoft Office PowerPoint.

### Cell-cell fusion

Cell-cell fusion was performed as previously described (6, 25, 30). 293T cells were co-transfected with spike plasmids and GFP. The cells were then detached using trypsin + 0.53 mM EDTA and co-cultured with either 293T-ACE2 or CaLu-3 cells. Cells were co-cultured for 6 h (293T-ACE2) or 4 h (CaLu-3) before fusion was imaged using a Leica DMi8 fluorescence microscope. The Leica X Applications Suite was used to quantify total areas of fusion by outlining areas of GFP fluorescence and calculating the area within these spaces. Only GFP-positive areas larger than 800 μm² were scored as fusion events, and the no-Spike condition was used to define background. Scale bars represent 750 µM. Three representative images were taken for each variant and used for quantification; one representative image was chosen for presentation.

### Spike protein surface expression detected by flow cytometry

Surface expression of spike was determined on 293T cells used to produce pseudotyped viruses. After the collection of virus 72 h post-transfection, cells were detached using PBS + 5 mM EDTA and then fixed in 3.7% formaldehyde. Cells were stained with an anti-S1 polyclonal antibody (Sino Biological, T62-40591, RRID:AB_2893171) and an anti-Rabbit-IgG FITC secondary antibody (Sigma, F9887, RRID:AB_259816). Flow cytometry data were collected using an Attune NxT flow cytometer and analyzed using FlowJo v10.8.1.

### Spike processing

Spike processing by furin was determined by lysing 293T cells producing pseudotyped viruses using RIPA buffer (Sigma Aldrich, R0278) plus protease inhibitor cocktails (Sigma, P8340). Samples were run on a 10% SDS-polyacrylamide gel and transferred onto a PVDF membrane. Blots were probed with anti-S2 (Sino Biological, T62-40590, RRID:AB_2857932), anti-S1 (Sino Bio, T62-40591, RRID:AB_2893171), anti-p24 (Abcam, ab63917; NIH ARP-1513), and anti-β-actin (Santa Cruz, Cat# sc-47778, RRID: AB_626632) antibodies, respectively. Secondary antibodies used were anti-Rabbit-IgG-HRP (Sigma, Cat#A9169, RRID:AB_258434) and anti-Mouse-IgG-HRP (Sigma, Cat#A5728, RRID:AB_258232). Chemiluminescence was determined by applying Immobilon Crescendo Western HRP substrate (Millipore, WBLUR0500) to the blots, followed by immediately reading on a GE Amersham Imager 600. NIH ImageJ (Bethesda, MD) was used to quantify S2/S and S1/S ratios based on relative band intensity.

### S1 shedding

HEK293T cells were transfected with spike expression constructs. Twenty-four hours after transfection, cells were treated with or without sACE2 (10 μg/mL) for 4 h at 37°C. Cell lysates and culture media were harvested. S1-containing cell culture media were incubated overnight with 10 μL of protein A/G beads (Santa Cruz, sc-2003) conjugated with a polyclonal anti-S1 antibody that recognizes epitopes conserved across all variants to precipitate the S1 subunit. Following immunoprecipitation, cell lysates and shed S1 were run on 10% SDS-PAGE, transferred to membranes, and probed with anti-S1 (Sino Biological, T62-40591, RRID:AB_2893171), anti-S2 (Sino Biological, T62-40590, RRID:AB_2857932), and anti-β-actin (Santa Cruz, Cat# sc-47778, RRID: AB_626632) antibodies, respectively. Anti-mouse-IgG-Peroxidase (Sigma, A5278) and anti-rabbit-IgG-HRP (Sigma, A9169) were used as secondary antibodies.

### Quantification and statistical analysis

All statistical analyses in this work were conducted using GraphPad Prism 10. NT_50_ values were calculated by least-squares fit non-linear regression. Error bars in Fig. 1C and D, 3C and D, 4B and E, 5A and B, and 6C, D, and G represent means ± standard errors. Error bars in Fig. 2A, C, and E and Fig. 5C, D, and E represent geometric means with 95% confidence intervals. Error bars in Fig. 2A, C, and E and Fig. 5C, D, and E represent means ± standard deviation. Statistical significance was analyzed using log10 transformed NT_50_ values to better approximate normality (Fig. 2A, C, and E and Fig. 5C, D, and E), and multiple groups comparisons were made using a one-way ANOVA with Bonferroni post-test. Cell-cell fusion was quantified using Leica X Applications Suite software (Fig. 3A and B and Fig. 6A and B). S processing was quantified by NIH ImageJ (Fig. 4C and D and Fig. 6E and F).
